# *Cortex Mori* extracts induce apoptosis and inhibit tumor invasion *via* blockage of the PI3K/AKT signaling in melanoma cells

**DOI:** 10.3389/fphar.2022.1007279

**Published:** 2022-10-19

**Authors:** Xin Hu, Kui Zhang, Guangzhao Pan, Yinggang Wang, Yue Shen, Cheng Peng, Longfei Deng, Hongjuan Cui

**Affiliations:** ^1^ State Key Laboratory of Silkworm Genome Biology, College of Sericulture, Textile and Biomass Sciences, Southwest University, Chongqing, China; ^2^ Cancer Center, Medical Research Institute, Southwest University, Chongqing, China; ^3^ Chongqing Engineering and Technology Research Center for Silk Biomaterials and Regenerative Medicine, Chongqing, China; ^4^ Southwest University Engineering Research Center for Cancer Biomedical and Translational Medicine, Chongqing, China

**Keywords:** melanoma, network pharmacology, anti-cancer natural product, molecular docking, *Cortex Mori*

## Abstract

Melanoma, the most aggressive and deadliest form of skin cancer, has attracted increased attention due to its increasing incidence worldwide. The *Cortex Mori* (CM) has long been used as a classical traditional Chinese medicine (TCM) to treat various diseases, including cancer. The bioactive components and underlying mechanisms, however, remain largely unknown. The current study aims to investigate the anti-melanoma effects of CM and potential mechanisms through combined network pharmacology and bioinformatic analyses, and validated by *in vitro* and *in vivo* experiments. We report here that CM has anti-melanoma activity both *in vitro* and *in vivo*. Furthermore, 25 bioactive compounds in CM were found to share 142 melanoma targets, and network pharmacology and enrichment analyses suggested that CM inhibits melanoma through multiple biological processes and signaling pathways, particularly the PI3K-AKT signaling inhibition and activation of apoptotic pathways, which were further confirmed by biochemical and histological examinations. Finally, partial CM-derived bioactive compounds were found to show anti-melanoma effects, validating the anti-melanoma potential of bioactive ingredients of CM. Taken together, these results reveal bioactive components and mechanisms of CM in inhibiting melanoma, providing them as potential anti-cancer natural products for the treatment of melanoma.

## Introduction

Melanoma, derived from the malignant transformation of melanocytes, is a highly aggressive and drug-resistant malignancy, with a 5-year survival rate of merely 29.8% (Huang and Zappasodi, 2022; Li et al., 2022). Currently, surgical excision is the most common clinical treatment for melanoma, followed by postoperative adjuvant treatments such as radiation or chemotherapy (Carr et al., 2020). However, these treatments usually elicit low response rates and severe side effects (Tyagi et al., 2022). As a result, developing new drugs or adjuvants with high activity and low toxicity for melanoma treatment is critical.

TCM has been increasingly utilized in Asia for thousands of years to treat a variety of illnesses (Xiang et al., 2019). TCM has recently gained widespread acceptance as an adjuvant therapy option following surgery, chemotherapy, radiotherapy, or other types of therapy due to its beneficial therapeutic outcomes for cancer patients (Chen et al., 2008). Furthermore, natural compounds derived from herbs are considered suitable drugs for cancer therapies due to their advantages of multi-targeting, efficacy, and low toxicity (Sayanta et al., 2019). CM, derived from the *Morus alba L.* (a mulberry tree), is commonly used as TCM for the alleviation of various diseases, such as cough (Yang et al., 2014), diabetes (Wang et al., 2020), inflammation (Carson et al., 2002), and cancers (Nam et al., 2002; Park et al., 2012; Guo et al., 2022). Modern pharmacological studies have revealed that the anti-lung cancer effect of CM extracts can induce cell apoptosis by inhibiting microtubule assembly (Carson et al., 2002). CM extracts also showed anti-tumor activity in colon cancer, leukemia, and hepatic cancer (Huang et al., 2011; Chen et al., 2017). Besides, previous studies have confirmed CM extracts possess anti-melanogenic and cytotoxic effects against mouse skin melanoma B16-F10 cells (Choe, 2011; Wu et al., 2018). However, the specific effective bioactive compounds and the underlying molecular mechanisms of CM remain unclear.

The complexity of various components, multiple targets, and synergistic interactions makes it difficult to explore the underlying mechanism of TCM (Ma et al., 2015). Network pharmacology, on the other hand, would systematically discover the interaction of the complex, diverse relationships among targets, drugs, diseases, and pathways, thereby providing a systematic approach to evaluate the feasibility and compatibility of TCM by delineating the detailed “multi-gene-multi-target-complex disease” multi-level interaction networks (Hopkins, 2007; Liu et al., 2022; Liu et al., 2022). Since its initial presentation in 2007, network pharmacology has been used extensively as a promising approach to TCM research (Hopkins, 2007).

In the present study, we investigated the potential bioactive compounds in CM using pharmacological indexes, and the anti-melanoma mechanisms of CM were determined using network pharmacology molecular docking and other bioinformatic analyses. Importantly, *in vitro* and *in vivo* experiments were carried out to validate the anti-cancer effects and underlying mechanisms of CM-derived bioactive ingredients in melanoma inhibition.

## Materials and methods

### Cell lines and reagents

Malignant melanoma cell lines A375 and MV3 were procured from the American Type Culture Collection (CRL-1619) and Beyotime (C6282), respectively. All cell lines were cultured in a humidified incubator with 5% CO_2_ at 37°C and maintained in high-glucose Dulbecco’s modified Eagle’s medium (DMEM; VivaCell, China) supplemented with 10% fetal bovine serum (FBS; BI, United States), 100 U/mL penicillin, and 100 μg/ml streptomycin. Sanggenone H (HY-N2607), Moracin O (HY-N3244), Kaempferol (HY-14590), and Mulberroside C (HY-N0620) were obtained from MCE (China). *Cortex Mori* was purchased from Tianjiang Pharmaceutical (China). Antibodies against human Tubulin (AF1216) were obtained from Beyotime (China), and antibodies against phospho-Akt (Thr308, 13038), BAX (2774), Bcl-2 (15,071), phospho-PI3 Kinase p85 (p-PI3K Tyr458, 17366), Cleaved Caspase-9 (9507), and Cleaved PARP (5625) were purchased from Cell Signaling Technology (CST).

### Characterization of bioactive ingredients in *cortex mori*

The bioactive compounds from CM were determined using the Traditional Chinese Medicine Systems Pharmacology Database and Analysis Platform (TCMSP, https://old.tcmsp-e.com/tcmsp.php), which comprises pharmacokinetic properties for natural compounds involving oral bioavailability (OB), drug-likeness (DL), intestinal epithelial permeability, blood-brain-barrier, aqueous solubility, etc., (Ru et al., 2014). Based on screening conditions with OB ≥ 30% and DL ≥ 0.18, around 25 active compounds were obtained.

### Target prediction of active ingredients in *cortex mori* and network construction

The protein targets of the active substances in CM were retrieved from the TCMSP database. GeneCard (https://www.genecards.org/, targets with relevance score ≥1 were screened) and OMIM (Online Mendelian Inheritance in Man, https://omim.org/search/advanced/geneMap) were used to collect melanoma-related target genes. Finally, target genes of CM for the treatment of melanoma were acquired by the R package “Venn”.

The cross-targets obtained above were entered into the search tool for the Retrieval of Interacting Genes (STRING) database (https://string-db.org) to construct a PPI network. 0.95 was taken as the minimum required interaction score, and the disconnected targets were removed (Abhimanyu et al., 2022; Pan et al., 2022). The PPI network was then analyzed and adjusted by Cytoscape-3.9.0 for further screening with median values of Betweenness (BC), Closeness (CC), Degree (DC), and Eigenvector (EC) using a plug-in of Cytoscape, CytoNCA.

The compound-target network was established using Cytoscape-3.9.0 to further analyze the molecular mechanism of CM in the therapy of melanoma. Compounds, targets, CM, and melanoma were shown as nodes in the graphical network, while the interactions were represented as edges.

### Enrichment analysis

The cross target gene names were transformed into entrezID by the R package “org.Hs.eg.db” and the Gene Ontology (GO) and Kyoto Encyclopedia of Genes and Genomes (KEGG) pathway analysis were conducted and visualized with the R packages “clusterProfiler”, “org.Hs.eg.db”, “enrichplot”, “ggplot2”, and “pathview” with adjusted *p*-values of <0.05.

### Molecular docking analysis

To further verify the binding capacity of bioactive compounds and potential targets, the core targets were chosen as receptors for molecular docking. The 2D structures of bioactive compounds were obtained from PubChem (https://pubchem.ncbi.nlm.nih.gov/) and imported into ChemBio3D 14.0 software to minimize energy and obtain 3D structures, which were further inputted into AutoDockTools-1.5.7 and saved in “pdbqt” format. The protein 3D structures were downloaded from the Protein Data Bank (PDB, http://www.rcsb.org/) and then imported into PyMOL-2.5.2 to remove organics, solvents, and ions. Subsequently, hydrogens were added by the AutoDockTools-1.5.7 software, and the resultant structure was saved in “pdbqt” format. The center grid box of the target protein was determined by AutoDockTools-1.5.7 based on the parameters as follows: 40 points in x-, y-, and z-dimension and 1.000 for spacing (angsrom). Finally, AutoDock Vinna-1.1.2 software was employed for molecular docking, and the results were visualized by PyMOL-2.5.2.

### Colony formation assay

A375 and MV3 cells were seeded in a 24-well plate at a density of 500 cells per well. Cells were cultured overnight before treatment with DMSO or different bioactive substances for 7 days. To count the colonies, the cell monolayer was rinsed twice with 1× PBS, fixed with 4% paraformaldehyde (PFA), and stained with 1% crystal violet solution. The number of colonies in each well was counted.

### EdU staining

Click-iT^®^ EdU Imaging Kits (Invitrogen, United States) were used for cell proliferative ability assay as described previously (Li et al., 2021). Approximately 2 × 10^4^ cells were seeded into 24-well cell culture plates and placed in an incubator overnight, and then incubated with DMSO or various bioactive compounds for 48 h. Then, the cells were incubated with 10 μM EdU (Sigma Aldrich, United States) for 40 min and fixed with 4% PFA for 15 min. The cells were then rinsed thrice with 1× PBS before being permeabilized for 2 hours with in 0.5% Triton X-100 in 5% bovine serum albumin (BSA). The cells were then incubated with Click-iT^®^ reaction cocktails at room temperature protected from light for 40 min. The nuclei were stained with DAPI for 20 min at room temperature. The cells were imaged by an inverted fluorescent microscope, and the number of cells that were positive for EdU in each field was calculated (Ji et al., 2022).

### Wound-healing assay

When the cells reach 100% confluency, linear wounds are carved using 200 µL pipette tips. After being washed thrice with 1× PBS, the cells were cultured in an FBS-free medium containing DMSO or various bioactive ingredients. After 30 h, the wounds were imaged and the closure rates were determined.

### Measurement of cell viability

The MV3 and A375 cells were seeded in a 96-well plate at a density of 1× 10^3^ cells per well. After 24 h, the cells were treated for another 48 h with DMSO or various doses of bioactive substances. The CCK8 assay was used to assess cell viability. The absorbance in each well was measured at 450 nm using a microplate reader (Thermo Fisher, United States).

### Cell apoptosis analysis

Cell apoptosis was determined using the cell apoptosis kits procured from Beyotime (C1062M, China). After 48 h of treatment with DMSO or CM, cells were harvested and washed thrice with 1 X PBS. Then cells were incubated with Annexin V-FITC and propidium at room temperature for 15 min in the dark according to the manufacturer’s protocol. Finally, the apoptotic cell ratio was detected using a flow cytometer and analyzed by FlowJo_v10.6.2 software.

### Western blotting

Total protein was extracted using RIPA (Beyotime, P0013K) with phenylmethanesulfonyl fluoride (PMSF) and phosphatase inhibitor (Abcam, ab201112), and the protein concentration was detected using a bicinchoninic acid (BCA) kit (ThermoFisher Scientific, 23227). 30 µg of sample protein per lane was added, and the loading volume for each sample was normalized using deionized water. As described previously, the protein was separated on 8%–15% SDS-PAGE gels and then transferred to polyvinylidene fluoride (PVDF) membranes. After a 2-h incubation with 5% BSA, the membranes were incubated with primary antibodies at 4°C overnight and washed three times with 1× TBST (10 min), followed by incubation with second antibodies at room temperature for 2 h (Zhang et al., 2018; Li et al., 2020). The relative expression of each target protein was measured using β- Tubulin as the reference.

### Quantitative real-time PCR

Total RNA was extracted and purified using RNAiso Plus (Takara, Japan) and was further used to synthesize the first-strand cDNA using the GoScriptTM Reverse Transcription System (Promega, United States). qRT-PCR was performed on a Roche LightCycler 96, using GoTaq^®^ qPCR master mix (Promega, United States) with the following reaction mixture: 2 µl cDNA template, 10 µl 2 × GoTaq®qPCR master mix, 7 µl nuclease-free water, and 0.5 µl forward and reverse primers. All qRT-PCR primers were obtained from PrimerBank (https://pga.mgh.harvard.edu/primerbank/) and are listed in Supplementary Table S1. Glyceraldehyde-3-phosphate dehy drogenase (GAPDH) served as an internal control. The 2-ΔΔCt method was used to determine the relative mRNA expression level (Zhang et al., 2017; Yang et al., 2020; Hu et al., 2021).

### Tumor xenograft model

BALB/c null mice were obtained from Gempharmatech Co., Ltd. (Chengdu, China) and raised in the specific pathogen-free (SPF) room. The Committee for Animal Protection and Utilization at Southwest University approved the study. Four-week-old mice were injected subcutaneously with A375 cells (5 × 10^5^) in the left flank. Mice were randomly allocated into the following two groups (*n* = 5) after a week: the control group (1× PBS) and the CM group. Each week, the tumor volume and the body weight of mice were measured. After that, mice were sacrificed, and tumors were collected for weight, H&E staining, and immunohistochemistry (IHC).

### Immunohistochemistry and H&E staining

Tumor samples were fixed, dehydrated, and embedded in paraffin for sections. The sections were stained with H&E or incubated in 0.3% H_2_O_2_ to block endogenous peroxidase for 20 min at room temperature. After blocking, the sections were incubated overnight with Ki67 or 4′, 6-diamidino-2-phenylindole. Finally, the sections were incubated with a biotinylated anti-rabbit antibody and observed under the microscope (Lian et al., 2022).

### TUNEL staining

Cellular apoptosis in tumor tissues was detected using the DAB (SA-HRP) Tunel Cell Apoptosis Detection Kit (G1507, Servicebio, China) by the manufacturer’s instructions. The sections were stained with Biotin-labeled dUTP (Biotin-dUTP) for 1 h at 37°C, then incubated with Streptavidin-HRP (SA-HRP) for 10 min at 37°C followed by 3,3′-diaminobenzidine (DAB). The sections were observed and imaged using microscopy, and the nuclei of apoptotic cells were stained brown.

### Statistical analysis

The data from all three biologically independent replicates are presented as mean ± SD. GraphPad Prism 8.0 was used to determine the significant differences between groups using the Student’s t-test. The asterisks indicate statistically significant differences (*p < 0.05 and **p < 0.01).

## Results

### CM exhibits anti-melanoma activity *in vitro* and *in vivo*

**FIGURE 1:**
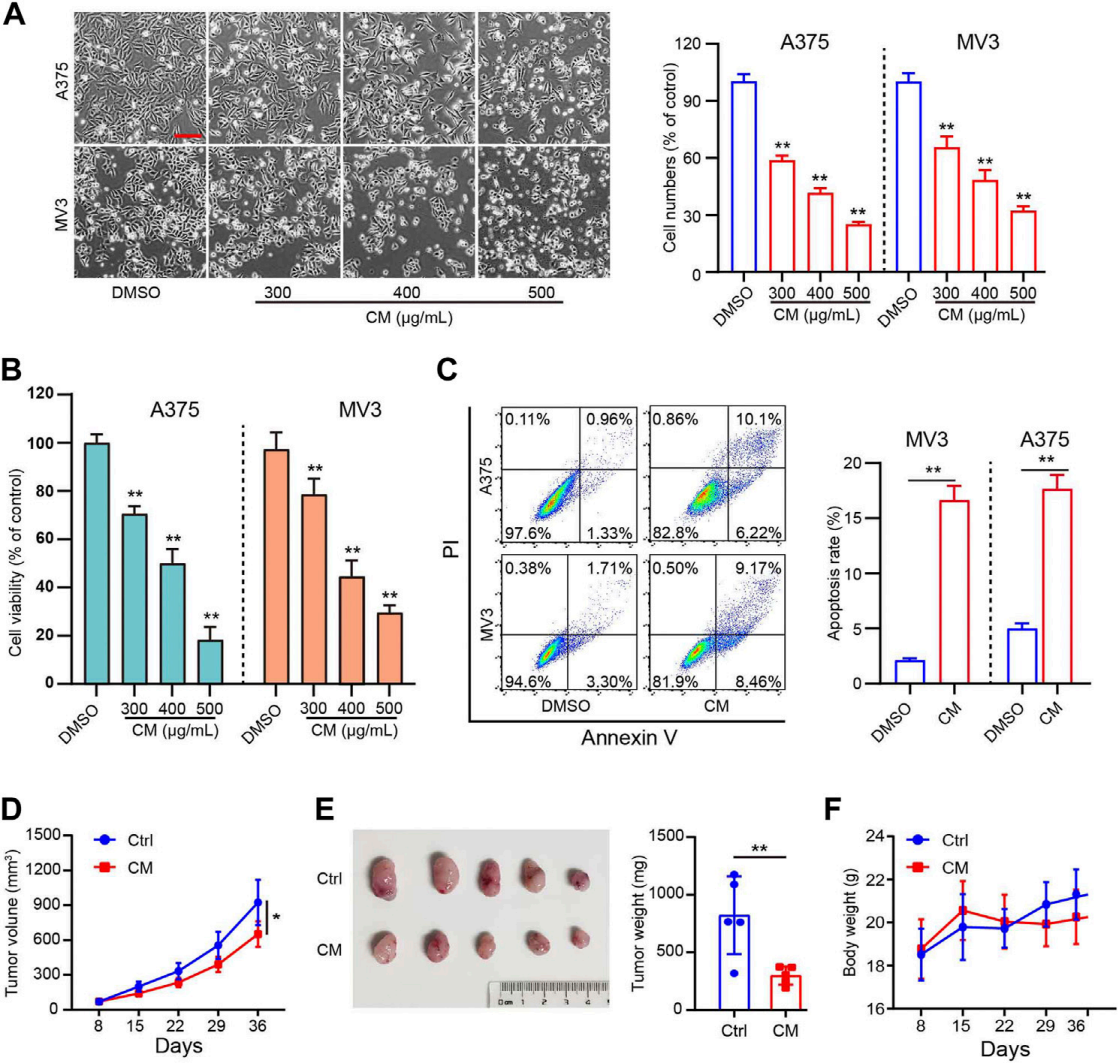
CM inhibits melanoma growth *in vitro* and *in vivo*. **(A)** Cell morphology of A375 and MV3 cells after 48 h of incubation with CM or DMSO and the percentage of cells in each group is represented by the histograms, with the control group being 100% of the cells. Scale bar: 1 mm. **(B)** Cytotoxicity of CM to melanoma carcinoma cell lines A375 and MV3. Cells were incubated with CM for 48 h, and the viability was determined by CCK8 assay. **(C)** A375 and MV3 cells were treated with CM for 48 h, and the apoptotic cells were evaluated and quantified by Annexin V/PI staining and flow cytometry. **(D)** Tumor growth curves of A375 tumor-bearing nude mice treated with control (*n* = 5) or CM (*n* = 5). **(E)** The effect of CM on tumor weight after resection. **(F)** The effect of CM on mice body weight. The asterisks indicate statistically significant differences (*p < 0.05 and **p < 0.01). Ctrl: control. CM: *Cortex Mori* extracts.

In the present study, *in vitro* experiments were first conducted to validate the anti-melanoma effects of CM. The A375 and MV3 cells exposed to CM changed their morphology and cell numbers dramatically (Figure 1A). The CCK8 assay revealed a significant dose-dependent decrease viability (Figure 1B). As shown in Figure 1C, CM treatment resulted in significant cell apoptosis revealed by flow cytometry. Furthermore, nude mice xenografted subcutaneously with A375 cells were used to test the anti-cancer effects of CM *in vivo*. CM treatment significantly reduced tumor growth compared to the control group (Figure 1D,E). It is worth mentioning that CM treatment had no significant effect on the body weight of tested mice (Figure 1F).

### Identification of bioactive compounds in *cortex mori*

To understand the underlying mechanism of CM against melanoma, bioactive compounds in CM were identified. A total of 194 ingredients were collected from TCMSP, of which 25 bioactive compounds were obtained under screening conditions with OB ≥ 30% and DL ≥ 0.18. These compounds are mostly flavonoids and phenols according to structural classification, and their detailed information is provided in Table 1. These bioactive ingredients were chosen for further investigation.

**TABLE 1:** Characteristics of active ingredients in *Mori Cortex*.

Molecule ID	Molecule Name	Molecular Weight	OB (%)	DL	Structural Classification
MOL000098	Quercetin	302.25	46.43	0.28	Flavonoids/Phenols
MOL000211	Mairin	456.78	55.38	0.78	Terpenoids
MOL000358	Beta-sitosterol	414.79	36.91	0.75	Steroids
MOL000422	Kaempferol	286.25	41.88	0.24	Flavonoids/Phenols
MOL001004	Pelargonidin	271.26	37.99	0.21	Flavonoids/Phenols
MOL001474	Sanguinarine	332.35	37.81	0.86	Alkaloids
MOL002514	Sexangularetin	316.28	62.86	0.3	Flavonoids/Phenols
MOL003758	Iristectorigenin (9CI)	330.31	71.55	0.34	Flavonoids/Phenols
MOL003856	Moracin B	286.3	55.85	0.23	Flavonoids/Phenols
MOL003857	Moracin C	310.37	82.13	0.29	Phenols
MOL003858	Moracin D	308.35	60.93	0.38	Phenols
MOL003860	Moracin F	286.3	53.81	0.23	Flavonoids/Phenols
MOL004912	Glabrone	336.36	52.51	0.5	Flavonoids/Phenols
MOL005043	Campest-5-en-3beta-ol	400.76	37.58	0.71	Steroids
MOL012681	Dimethyl (methylenedi-4,1- phenylene) biscarbamate	314.37	50.84	0.26	lipids
MOL012686	7-methoxy-5,4'-dihydroxyflavanonol	302.3	51.72	0.26	Alcohols/Flavonoids
MOL012689	cyclomulberrochromene	418.47	36.79	0.87	Flavonoids/Phenols
MOL012692	Kuwanon D	422.51	31.09	0.8	Flavonoids/Phenols
MOL012714	Moracin A	286.3	64.39	0.23	Phenols
MOL012719	Moracin O	326.37	62.33	0.44	Phenols
MOL012735	Mulberroside C	326.37	71.39	0.46	Phenols
MOL012753	Sanggenone F	354.38	62.42	0.54	Flavonoids/Phenols
MOL012755	Ssanggenone H	354.38	37.5	0.53	Flavonoids/Phenols
MOL012760	Sanggenone M	436.49	68.29	0.85	Flavonoids/Phenols
MOL012800	3,5,7-trihydroxy-2-(3- hydroxyphenyl) chromone	286.25	59.71	0.24	Flavonoids/Phenols

### Targets identification and PPI network establishment

**FIGURE 2:**
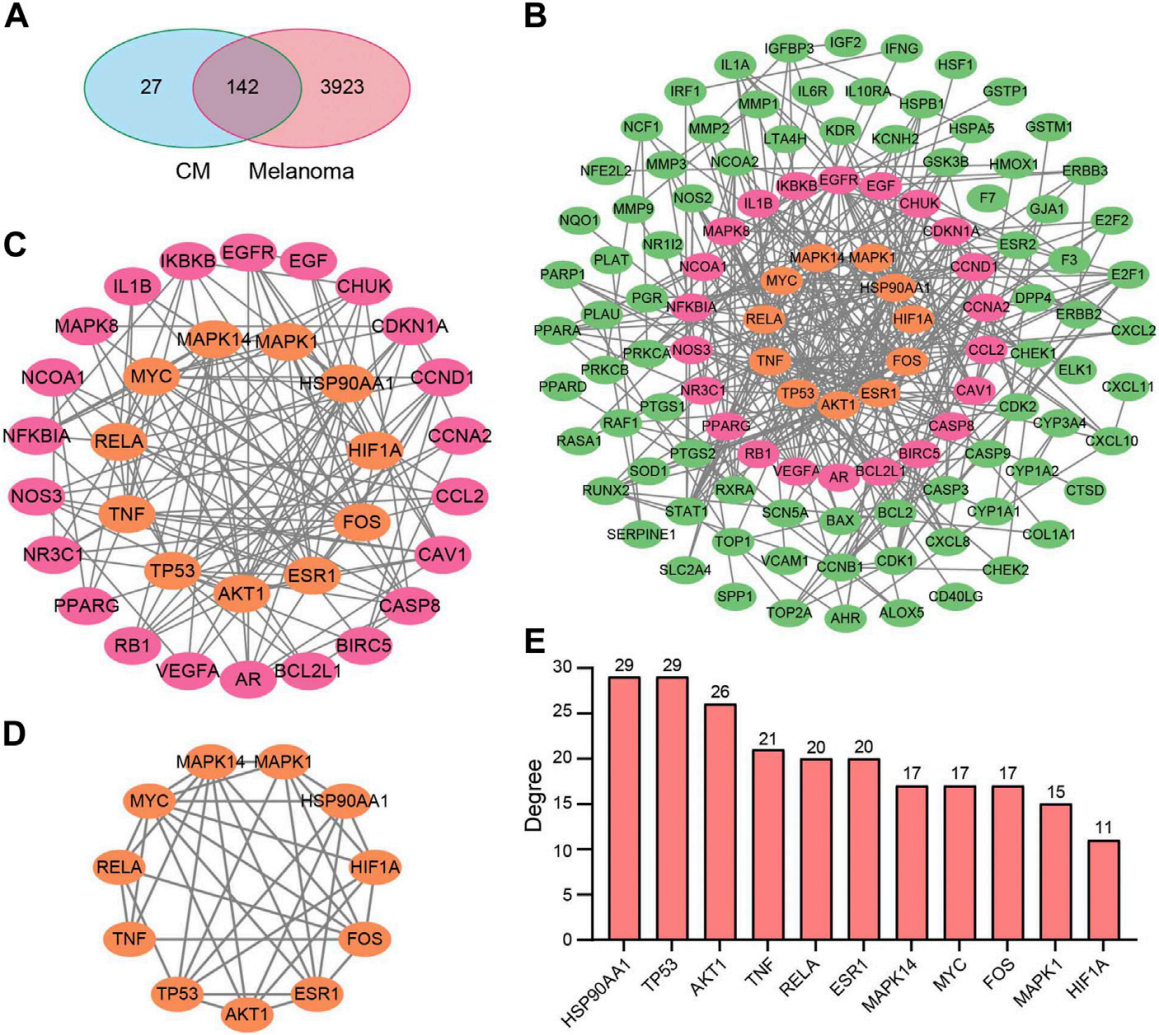
Potential target genes and PPI network map of CM treatment for melanoma. **(A)** Venn diagram of the potential targets of CM for the treatment of melanoma. **(B)** The PPI network map of 49 target genes. **(C)** The hub genes from **(B)** were screened using CytoNCA, and **(D)** 11 top hub genes were further screened from **(C)** using CytoNCA. **(E)** The degree value of the 11 top hub genes. CM: *Cortex Mori* extracts.

After removing duplicates, 169 target genes of the above 25 bioactive compounds were acquired from the source of DrugBank on TCMSP. In contrast, 4065 unique melanoma-related targets were obtained from the GeneCard and OMIM databases. 142 potential targets of CM for the treatment of melanoma were obtained (Figure 2A), which were listed in Table 2.

**TABLE 2:** Potential target genes of bioactive compounds in *Mori Cortex* for melanoma.

NO.	Symbol	Entrez ID	NO.	Symbol	Entrez ID	NO.	Symbol	Entrez ID	NO.	Symbol	Entrez ID
1	AR	367	37	AKR1B1	231	73	CYP3A4	1576	109	NQO1	1728
2	PTGS2	5743	38	MMP3	4314	74	CYP1A2	1544	110	PARP1	142
3	CA2	760	39	RELA	5970	75	CAV1	857	111	AHR	196
4	ADRB2	154	40	EGFR	1956	76	MYC	4609	112	SLC2A4	6517
5	DPP4	1803	41	AKT1	207	77	F3	2152	113	CXCL11	6373
6	LTA4H	4048	42	VEGFA	7422	78	GJA1	2697	114	CXCL2	2920
7	SCN5A	6331	43	CCND1	595	79	CYP1A1	1543	115	CHEK2	11200
8	NCOA2	10499	44	BCL2L1	598	80	ICAM1	3383	116	CLDN4	1364
9	HSP90AA1	3320	45	FOS	2353	81	IL1B	3553	117	PPARA	5465
10	ESR1	2099	46	CDKN1A	1026	82	CCL2	6347	118	PPARD	5467
11	PRSS1	5644	47	EIF6	3692	83	SELE	6401	119	HSF1	3297
12	CDK2	1017	48	PLAU	5328	84	VCAM1	7412	120	CXCL10	3627
13	CCNA2	890	49	MMP2	4313	85	PTGER3	5733	121	CHUK	1147
14	NOS2	4843	50	MMP9	4318	86	CXCL8	3576	122	SPP1	6696
15	PTGS1	5742	51	MAPK1	5594	87	PRKCB	5579	123	RUNX2	860
16	F7	2155	52	IL10RA	3587	88	BIRC5	332	124	RASSF1	11186
17	KDR	3791	53	EGF	1950	89	NOS3	4846	125	E2F1	1869
18	ESR2	2100	54	RB1	5925	90	HSPB1	3315	126	E2F2	1870
19	NCOA1	8648	55	TNF	7124	91	IL2RA	3559	127	ACP3	55
20	KCNH2	3757	56	IL6R	3570	92	NR1I2	8856	128	CTSD	1509
21	RXRA	6256	57	TP53	7157	93	CYP1B1	1545	129	IGFBP3	3486
22	PGR	5241	58	ELK1	2002	94	CCNB1	891	130	IGF2	3481
23	SLC6A4	6532	59	NFKBIA	4792	95	PLAT	5327	131	CD40LG	959
24	OPRM1	4988	60	ODC1	4953	96	THBD	7056	132	IRF1	3659
25	BCL2	596	61	TOP1	7150	97	SERPINE1	5054	133	ERBB3	2065
26	BAX	581	62	RAF1	5894	98	COL1A1	1277	134	HK2	3099
27	CASP9	842	63	SOD1	6647	99	IFNG	3458	135	RASA1	5921
28	CASP3	836	64	MMP1	4312	100	ALOX5	240	136	GSTM1	2944
29	CASP8	841	65	HIF1A	3091	101	IL1A	3552	137	NR3C2	4306
30	PRKCA	5578	66	STAT1	6772	102	MPO	4353	138	NR3C1	2908
31	MAP2	4133	67	RUNX1T1	862	103	TOP2A	7153	139	IKBKB	3551
32	PPARG	5468	68	CDK1	983	104	NCF1	653361	140	MAPK8	5599
33	MAPK14	1432	69	HSPA5	3309	105	ABCG2	9429	141	PPP3CA	5530
34	GSK3B	2932	70	ERBB2	2064	106	HAS2	3037	142	AKR1C3	8644
35	CHEK1	1111	71	ACACA	31	107	GSTP1	2950			
36	ACHE	43	72	HMOX1	3162	108	NFE2L2	4780			

Subsequently, the PPI network was constructed by the STRING database and visualized by Cytoscape based on these 142 potential targets, which include 114 nodes and 367 edges (Figure 2B). Thereafter, the CytoNCA screen resulted in a network with 33 nodes and 136 edges based on median values of BC, DC, CC, and EC (Figure 2C). Finally, based on further above-mentioned screening, the core network with 11 nodes and 36 edges was acquired (Figure 2D). These hub genes include MAPK1, MAPK14, AKT1, TNF, HSP90AA1, TP53, RELA, MYC, ESR1, FOS, and HIF1A, and most of them have a degree in the PPI network no less than 15 (Figure 2E).

### Potential anti-melanoma mechanisms of *cortex mori* revealed by network pharmacology and enrichment analysis

**FIGURE 3:**
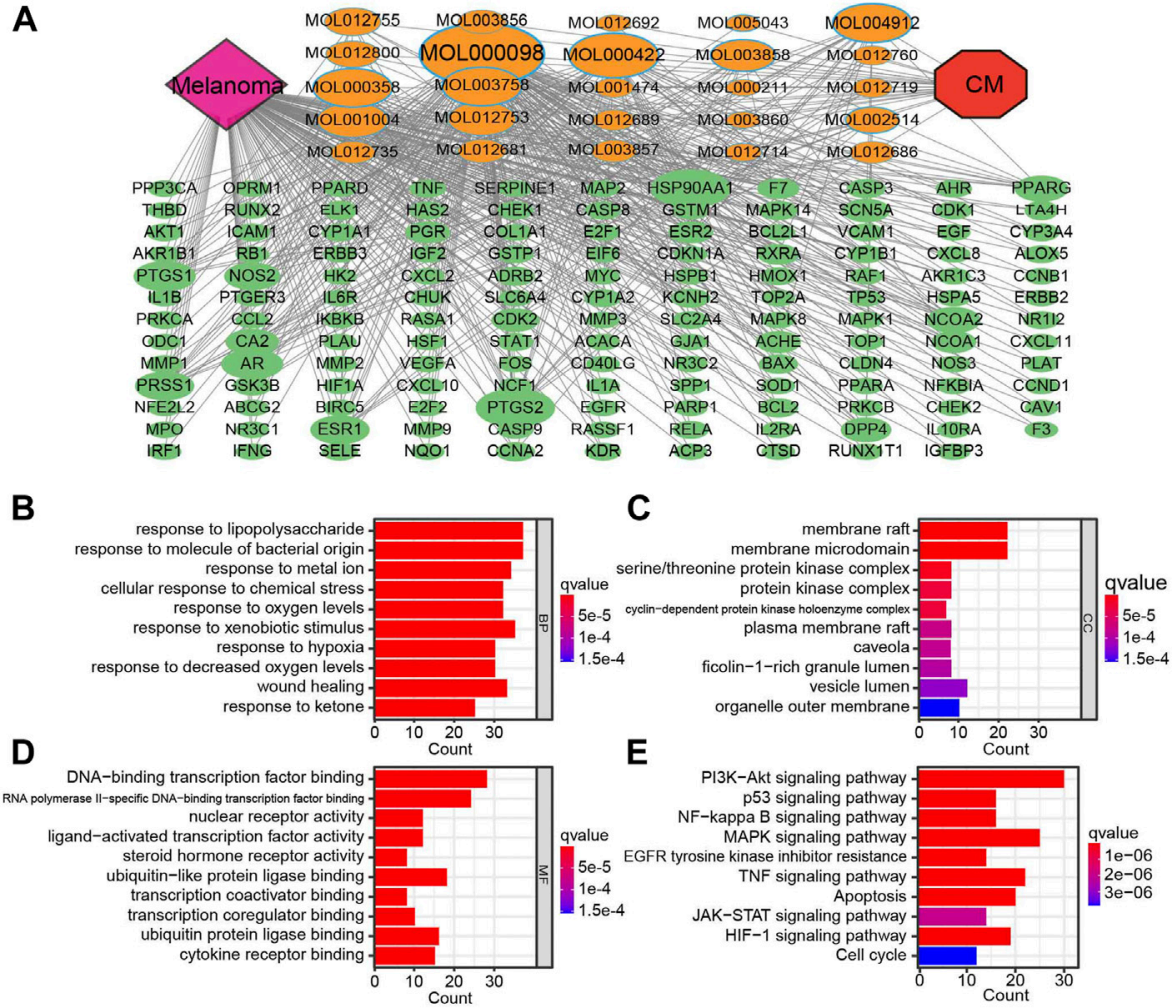
The compound-target network for CM on melanoma and enrichment analysis. **(A)** The purple node represents melanoma. The red node represents CM. The yellow nodes represent candidate active compounds, and the dark green nodes represent potential protein targets. The edges represent the interactions between them, and node sizes are proportional to their degree except for CM and melanoma. The gray connecting lines indicate that each node is interconnected. The top 10 GO terms in **(B)** biological process (BP), **(C)** cellular composition (CC), **(D)** and molecular function (BF) are displayed based on *p* < 0.05. **(E)** KEGG analysis for potential targets. The enriched signaling pathways of potential target genes. The Y-axis represents significant KEGG pathways, and the X-axis represents the count of targets. The gradient of color represents the adjusted *p*-value. CM: *Cortex Mori* extracts.

The CM–Active Ingredients–Targets–Melanoma network was constructed by Cytoscape, which has 169 nodes and 488 edges, demonstrating that the multi-component, multi-target, and multi-channel therapeutic properties of TCM (Figure 3A). After converting gene symbols into entrezIDs, 142 potential targets of 25 bioactive ingredients in CM for melanoma therapy were collected for GO enrichment and KEGG pathway enrichment analysis (Table 2). Go analysis revealed that the biological processes (BP) such as response to oxygen levels, response to hypoxia, and response to decreased oxygen levels, cellular components (CC) like serine/threonine protein kinase complex, protein kinase complex, and cyclin-dependent protein kinase holoenzyme complex, and molecular function (MF) such as DNA-binding transcription factor binding, ubiquitin-like protein ligase binding, and ubiquitin protein ligase binding may be potential anti-cancer mechanisms for CM for treatment of melanoma (Figures 3B–D). The KEGG pathway enrichment analysis (*p* < 0.05) demonstrated that CM may affect various signaling pathways closely related to carcinogenesis, including the PI3K-AKT signaling pathway, MAPK signaling pathway, TNF signaling pathway, apoptosis, and others (Figure 3E). Additionally, the PI3K-AKT signaling pathway was selected for annotation due to the significance of the enrichment, where many key factors are targets of bioactive ingredients in CM (Supplementary Figure S1).

### Active components of *cortex mori* target key genes in melanoma

**FIGURE 4:**
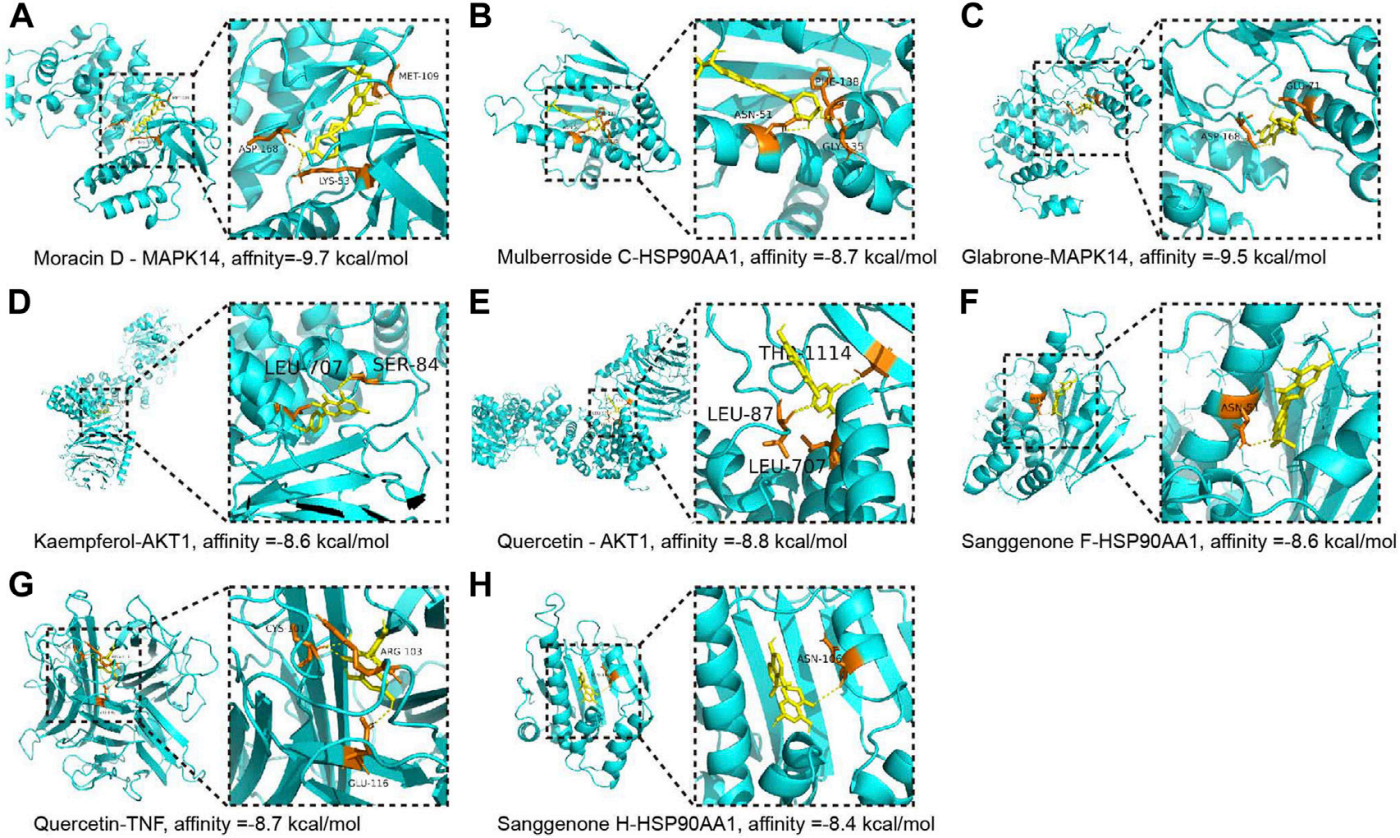
The molecular docking between the hub targets and bioactive compounds. **(A)** The 3D map of the binding of Moracin D and MAPK14, affinity = −9.7 kcal/mol; **(B)** The 3D map of the binding of Mulberroside C and HSP90AA1, affinity = −8.7 kcal/mol; **(C)** The 3D map of the binding of Glabrone and MAPK14, affinity = −9.5 kcal/mol; **(D)** The 3D map of the binding of kaempferol and AKT1, affinity = −8.6 kcal/mol; **(E)** The 3D map of the binding of quercetin and AKT1, affinity = −8.8 kcal/mol; **(F)** The 3D map of the binding of Sanggenone F and HSP90AA1, affinity = −8.6 kcal/mol; **(G)** The 3D map of the binding of quercetin and TNF, affinity = −8.7 kcal/mol; **(H)** The 3D map of the binding of Sanggenone H and HSP90AA1, affinity = −8.4 kcal/mol.

To reveal the candidate ingredients targeting melanoma-related proteins, 11 hub targets in the core PPI network were selected for molecular docking with corresponding active components in CM (Figure 2D). Lower binding energy (affinity) suggests a more potent and stable interaction between the compound and receptor, while binding energy of < −7.0 kcal mol−1 indicates better binding activity (Saraswati et al., 2019). According to docking results, three pairs share binding energy less than < −9.0 kcal mol^−1^, and the majority of molecular docking results have binding affinity less than −7.0 kcal mol^−1^, demonstrating that the active components of CM have good binding capacity toward melanoma target proteins (Table 3). The docking diagram of eight pairs of bioactive components and hub targets with higher affinity is shown in Figure 4, and the bioactive components include Moracin D, Quercetin, Glabrone, Mulberroside C, Kaempferol, Sanggenone F, and Sanggenone H.

**TABLE 3:** The results of molecular docking.

Chem	Mol ID	PDB	Receptor	Best affinity (kcal/mol)
Moracin D	MOL003858	6QE1	MAPK14	−9.7
quercetin	MOL000098	1A02	FOS	−9.5
Glabrone	MOL004912	6QE1	MAPK14	−9.5
quercetin	MOL000098	5WBL	AKT1	−8.8
quercetin	MOL000098	1A8M	TNF	−8.7
mulberroside C	MOL012735	1BYQ	HSP90AA1	−8.7
kaempferol	MOL000422	5WBL	AKT1	−8.6
sanggenone F	MOL012753	1BYQ	HSP90AA1	−8.6
sanggenone H	MOL012755	1BYQ	HSP90AA1	−8.4
kaempferol	MOL000422	1A8M	TNF	−8.3
quercetin	MOL000098	6WQX	TP53	−8.3
Moracin D	MOL003858	1A52	ESR1	−8.2
Moracin D	MOL003858	1BYQ	HSP90AA1	−8.2
mulberroside C	MOL012735	1A52	ESR1	−8
sanggenone F	MOL012753	1A52	ESR1	−8
quercetin	MOL000098	1H2K	HIF1A	−8
moracin O	MOL012719	1A52	ESR1	−7.9
moracin O	MOL012719	1BYQ	HSP90AA1	−7.8
quercetin	MOL000098	7E75	MAPK1	−7.8
Moracin C	MOL003857	1BYQ	HSP90AA1	−7.7
Moracin F	MOL012753	1BYQ	HSP90AA1	−7.7
Iristectorigenin (9CI)	MOL003758	6QE1	MAPK14	−7.7
Moracin A	MOL012714	1BYQ	HSP90AA1	−7.6
Sexangularetin	MOL002514	1BYQ	HSP90AA1	−7.6
sanggenone H	MOL012755	1A52	ESR1	−7.5
Moracin B	MOL003856	1BYQ	HSP90AA1	−7.5
quercetin	MOL000098	1BYQ	HSP90AA1	−7.5
Glabrone	MOL004912	1A52	ESR1	−7.4
sanggenone M	MOL012760	1A52	ESR1	−7.4
Moracin C	MOL003857	1A52	ESR1	−7.3
Iristectorigenin (9CI)	MOL003758	1BYQ	HSP90AA1	−7.3
pelargonidin	MOL001004	1BYQ	HSP90AA1	−7.2
kaempferol	MOL000422	1BYQ	HSP90AA1	−7.2
Iristectorigenin (9CI)	MOL003758	1A52	ESR1	−7.1
Moracin B	MOL003856	1A52	ESR1	−7.1
beta-sitosterol	MOL000358	1BYQ	HSP90AA1	−6.8
kaempferol	MOL000422	5XNX	RELA	−6.3
quercetin	MOL000098	5XNX	RELA	−6.1
quercetin	MOL000098	1A93	MYC	−5.6

### *Cortex mori* induce the PI3K/AKT pathway inhibition and apoptosis

**FIGURE 5:**
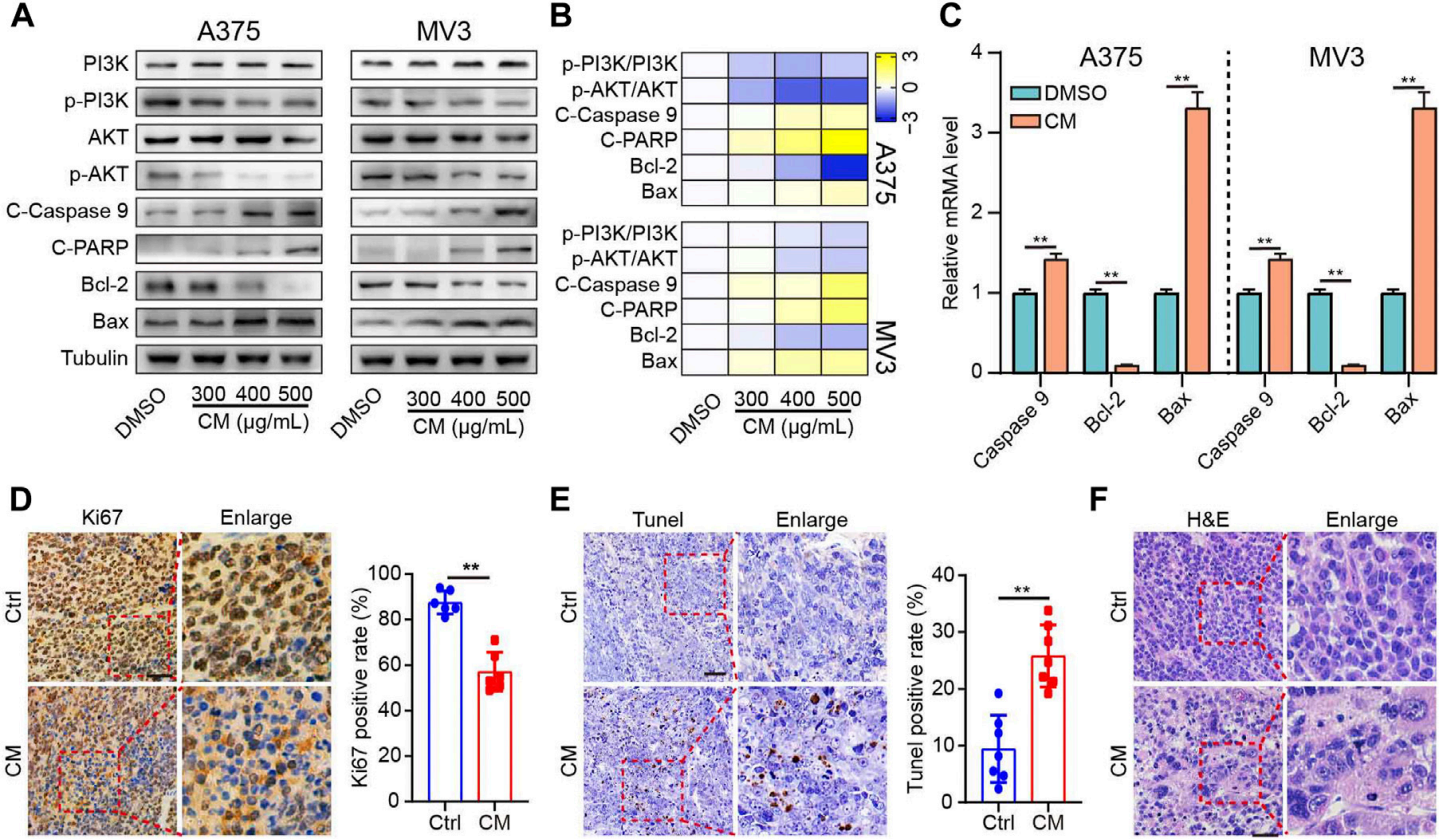
CM inhibits the growth by inducing apoptosis of melanoma cells and inhibiting the PI3K/AKT pathway. **(A)** The western blotting was performed on the A375 (left) and MV3 cells (right) against PI3K, p-PI3K, AKT, p-AKT, cleaved Caspase 9 (C-Caspase 9), cleaved PARP (C-PARP), Bcl- 2, and Bax. **(B)** Heat map visualization of Log_2_ [relative protein level] in panel **(A)**. **(C)** CM affected mRNA expression of Caspase 9, Bcl2, and Bax in A375 and MV3 cells analyzed by quantitative RT-PCR. **(D)** Ki67 immunohistochemistry staining in tumor sections and the percent of Ki67 positive cells were calculated as the fraction of Ki67 positive cells compared to the total number of cells. **(E)** The effect of CTFs on apoptosis of tumor tissue by TUNEL. **(F)** Histopathological examination of H&E staining in tumor sections. The asterisks indicate statistically significant differences (*p < 0.05 and **p < 0.01). Ctrl: control. CM: *Cortex Mori* extracts.

To further verify the underlying anti-melanoma mechanism of CM discovered through network pharmacology, protein and mRNA expression levels related to the PI3K/AKT pathway and apoptosis were investigated. The western blotting results showed that CM inhibited the protein expression of phospho-PI3K and phospho-AKT, two critical nodes in the PI3K/AKT pathway. Furthermore, both pro-apoptotic protein proteins (Cleaved caspase 9, Cleaved PARP, Bax) and mRNA (Caspase 9, Bax) levels were significantly increased in a dose-dependent manner in melanoma cells, whereas anti-apoptotic protein and mRNA level of Bcl2 was decreased sharply (Figure 5A–C). Meanwhile, the Ki67 staining results revealed that the CM-treated group had lower Ki67 protein levels than the control group (Figure 5D). TUNEL staining showed that CM treatment induced cell apoptosis in tumor tissues (Figure 5E). Furthermore, histological changes were observed by H&E staining, and CM treatment significantly reduced the number of nuclei in each field of view (Figure 5F).

#### *Cortex mori*-derived bioactive ingredients inhibited melanoma cell growth *in Vitro*

**FIGURE 6:**
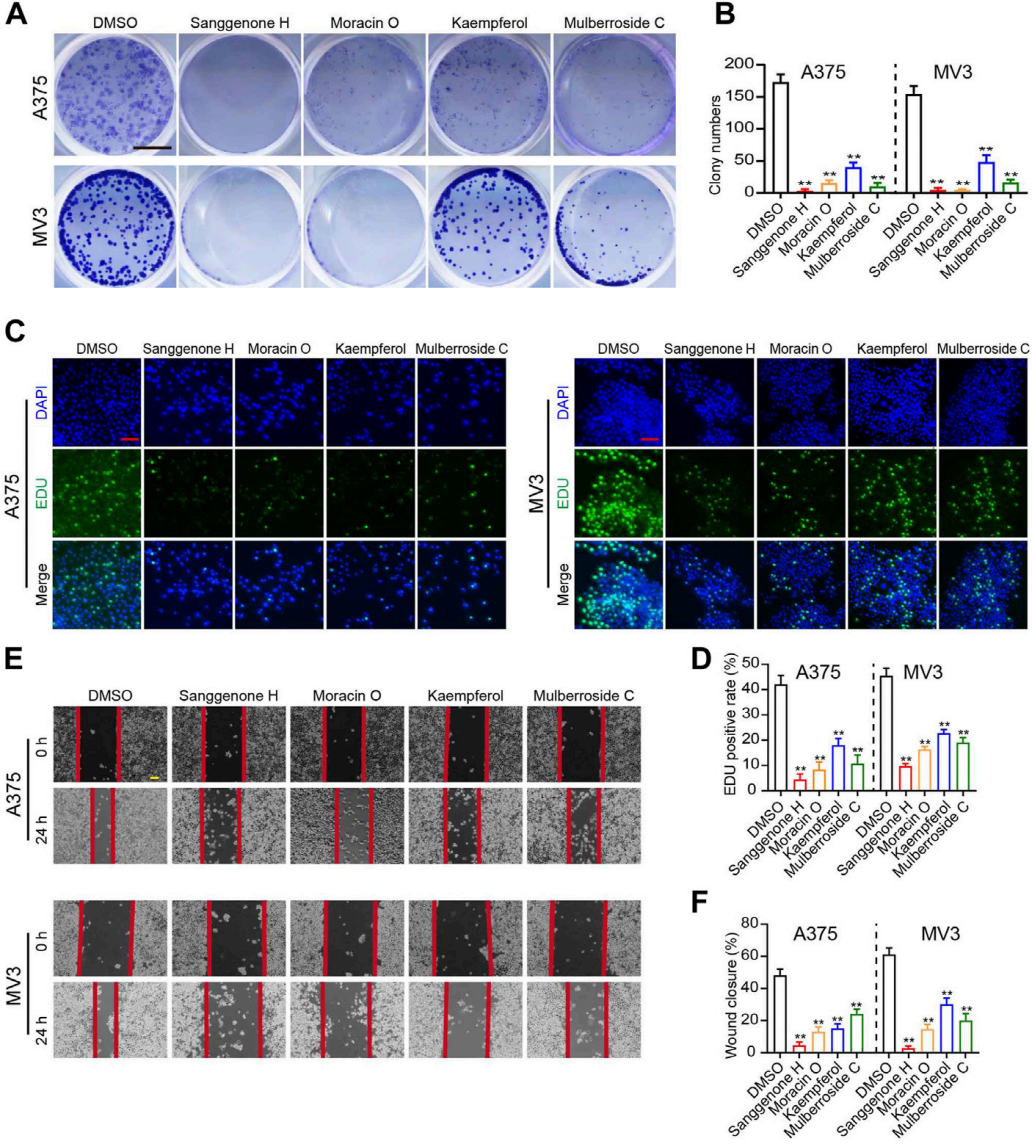
Anti-tumor effects of partial bioactive compounds extracted from CM on melanoma cells. **(A)** Colony formation assays and **(B)** Quantification of clones on A375 and MV3 cells treated with various bioactive compounds from CM for 7 days. DMSO was used as a control. The cells were stained with crystal violet staining solution. The scale bar indicates 5 mm. **(C)** Images and **(D)** quantification of EdU-positive A375 and MV3 cells treated with various bioactive compounds for 48 h. DMSO was used as a control. Scale bar = 50 μm. **(E)** The wounds on cell layers under the incubation of various bioactive compounds or DMSO were observed at 30 h, and **(F)** the closure rates were calculated. Scale bar = 100 μm. The asterisks indicate statistically significant differences (*p < 0.05 and **p < 0.01).

In the present study, four bioactive compounds derived from CM were selected to validate the anti-melanoma efficacy of bioactive ingredients in CM. A375 and MV3 cells exhibited significantly sliced colony formation ability when exposed to these bioactive compounds (Figure 6A–B). Furthermore, in an EdU staining assay, these bioactive substances significantly decreased the percentage of EdU-positive cells as compared to the control (Figure 6C,D). In addition, A375 and MV3 cells treated with bioactive compounds extracted from CM showed a significant impairment in migratory abilities (Figure 6E,F).

## Discussion

Melanoma, a highly metastatic tumor, is the leading cause of death from skin cancer (Tong and Young, 2014). Melanoma is largely fatal, though surgical resection of early-stage melanoma is frequently curative (Sykes et al., 2016; Du et al., 2020). Therefore, the discovery of effective natural compounds against melanoma may have important public health implications. Meanwhile, various signaling pathways and many molecular targets are characterized to be involved in melanoma progression (Li et al., 2022). Network pharmacology emphasizes multiple signaling pathway modulation, which increases drug therapeutic impact while decreasing toxic and side effects, making it a powerful method for the mechanistic study of complicated TCM against melanoma (Zuo et al., 2018). The main purpose of this study is to decipher the mechanism of CM for melanoma treatment through the application of network pharmacology and experimental verification.

In the present study, 25 bioactive compounds were identified from CM (Table 1). They were considered pharmacokinetically active under screen conditions with OB ≥ 30% and DL ≥ 0.18, indicating that they possessed essential pharmacokinetic properties for compounds to reach systemic circulation (Xu et al., 2012; Tao et al., 2013). Previous studies have shown that mulberry extracts have good anti-tumor activities. For instance, Sanggenon C has been reported to inhibit colon cancer cell proliferation and induce apoptosis by increasing reactive oxygen species, decreasing nitric oxide levels, and activating the mitochondrial apoptosis pathway (Chen et al., 2017). Anti-tumor properties of Sanggenon C have also been described in leukemia by inducing cell cycle arrest and cell death (Huang et al., 2011). Quercetin has anti-tumor activity in a variety of cancers, including non-small cell lung cancer, colorectal cancer, and others (Li et al., 2022; Liu et al., 2022). Kaempferol also has anti-tumor properties due to its modulation of the Akt/mTOR signaling and FAK pathways (Hung et al., 2017; Wang et al., 2021). In this study, four bioactive compounds derived from CM significantly inhibit melanoma cell proliferation and migration (Figure 6). These results suggest that CM-derived bioactive compounds, such as Moracin B, moracin C, Moracin D, Sanggenone F, Sanggenone H, and Sanggenone M, may have good anti-tumor properties that merit further investigations.

To determine the mechanism underlying the therapeutic effect of CM on melanoma, network pharmacology was used in this study, which revealed a core network containing 11 proteins (Figure 2). These proteins are well-known to be therapeutic targets for melanoma. AKT1, for instance, plays a key role in regulating cell survival, insulin signaling, angiogenesis, and tumor formation (Ronnstrand, 2004; Jia et al., 2021). Moreover, it has been shown that AKT1 promotes melanoma brain metastasis. Therefore, it could be a promising therapeutic target (Kircher et al., 2019). MYC and MAPK1 are involved in cell cycle progression, apoptosis, and cellular transformation, and their over-expression is common in melanoma (Savoia et al., 2019; Adam et al., 2020; Yang et al., 2022). The results of molecular docking verification revealed that CM-derived bioactive compounds had a high affinity for these targets (Figure 4; Table 3). These results suggest that CM could be applied in the alleviation and treatment of melanoma through the regulation of multiple targets by its bioactive ingredients.

GO and KEGG analysis showed that multiple molecular functions, biological processes, cellular components, and pathways were implicated in the potential treatment targets of CM for melanoma, embodying the TCM with characteristics of multiple targets and multiple pathways (Fu et al., 2022). Interestingly, multiple pieces of evidence in this study indicate that the HIF-1 signaling pathway, a master regulator of oxygen homeostasis, may be an important mechanism for CM in melanoma treatment. According to GO analysis, multiple molecular functions, such as response to oxygen levels, response to hypoxia, and response to decreased oxygen levels, point to oxygen levels (Figure 3). Furthermore, HIF1A was also discovered in the core network (Figure 2D), and quercetin exhibited excellent targeting ability to HIF1A (Saraswati et al., 2019), with the best affinity equal to -8.0 kcal/mol (Table 3). These findings are backed up by KEGG analysis (Figure 4).

Additionally, when exposed to CM, melanoma cells displayed significant proliferation inhibition and apoptosis induction, and expression levels related to apoptosis and the PI3K/AKT pathway were markedly affected (Figure 1; Figure 5). Previous studies have shown that the PI3K/AKT signaling pathway is an essential node in cancer cells that controls cell growth, migration, proliferation, and metabolism, and targeting the oncogenic PI3K/AKT signaling pathway is currently thought to be an extremely promising strategy for melanoma intervention (Shen et al., 2022; Vasan and Cantley, 2022). Apoptosis is crucial in tumorigenesis, development, and drug resistance (Braicu et al., 2022). Suppression of the PI3K/AKT pathway induces cell apoptosis *via* a variety of mechanisms, including regulation of Bcl-2 family members’ activities and activation of members of the caspase family of proteases (Kircher et al., 2019). Our results suggest that CM may inhibit melanoma cell proliferation and migration while inducing apoptosis *via* inhibition of the PI3K/Akt signaling pathway, providing a mechanistic foundation for exploiting CM and CM-derived bioactive components in melanoma treatment.

## Conclusion

The potential mechanisms of CM on melanoma were systematically investigated using a network pharmacology approach and experimental validation. A total of 25 bioactive compounds were determined in CM, sharing 142 melanoma targets. GO and KEGG pathway enrichment analysis were used to investigate biological functions and signaling pathways. Molecular docking confirmed the ability of bioactive compounds to bind to core targets. *In vivo* experiments revealed that bioactive compounds derived from partial CM-derived bioactive compounds had excellent anti-cancer effects on proliferation and migration. In addition, our results show that CM may inhibit the growth and induce apoptosis of melanoma cells through the PI3K/Akt pathway, supporting the results obtained from network pharmacology. These findings provide the anti-tumor components and mechanisms of CM on melanoma. Our results also suggest that the bioactive ingredients in CM may have anti-cancer properties that warrant further investigation. Meanwhile, the precise mechanisms and side effects of these active ingredients need to be investigated further.

## Data Availability

The original contributions presented in the study are included in the article/Supplementary Materials, further inquiries can be directed to the corresponding authors.
