# Supplementary material for: Cortex Mori extracts induce apoptosis and inhibit tumor invasion via blockage of the PI3K/AKT signaling in melanoma cells
Source: Front Pharmacol. 2022 Oct 19;13:1007279. doi: 10.3389/fphar.2022.1007279 (PMC9627489; doi:10.3389/fphar.2022.1007279)
Supplement: Supplementary file 1 [file Table1.docx]

**Table S1**. Primers used in this study

| Gene name | Primer name | Annealing Temperature (^◦^C) | Sequence (5′–3′) |
| --- | --- | --- | --- |
| PIK3R1 | PIK3R1-F | 54.41 | ACCACTACCGGAATGAATCTCT |
|  | PIK3R1-R | 54.43 | GGGATGTGCGGGTATATTCTTC |
| AKT1 | AKT1-F | 53.14 | AGCGACGTGGCTATTGTGAAG |
|  | AKT1-R | 55.61 | GCCATCATTCTTGAGGAGGAAGT |
| Caspase 9 | CASP9-F | 55.61 | CTTCGTTTCTGCGAACTAACAGG |
|  | CASP9-R | 62.00 | GCACCACTGGGGTAAGGTTT |
| Bcl2 | Bcl2-F | 62.00 | GGTGGGGTCATGTGTGTGG |
|  | Bcl2-R | 54.45 | CGGTTCAGGTACTCAGTCATCC |
| Bax | Bax-F | 53.16 | CCCGAGAGGTCTTTTTCCGAG |
|  | Bax-R | 53.14 | CCAGCCCATGATGGTTCTGAT |
| GAPDH | GAPDH-F | 53.14 | GGAGCGAGATCCCTCCAAAAT |
|  | GAPDH-R | 55.61 | GGCTGTTGTCATACTTCTCATGG |
