# Supplementary figures and images for: Cortex Mori extracts induce apoptosis and inhibit tumor invasion via blockage of the PI3K/AKT signaling in melanoma cells
Source: Front Pharmacol. 2022 Oct 19;13:1007279. doi: 10.3389/fphar.2022.1007279 (PMC9627489; doi:10.3389/fphar.2022.1007279)

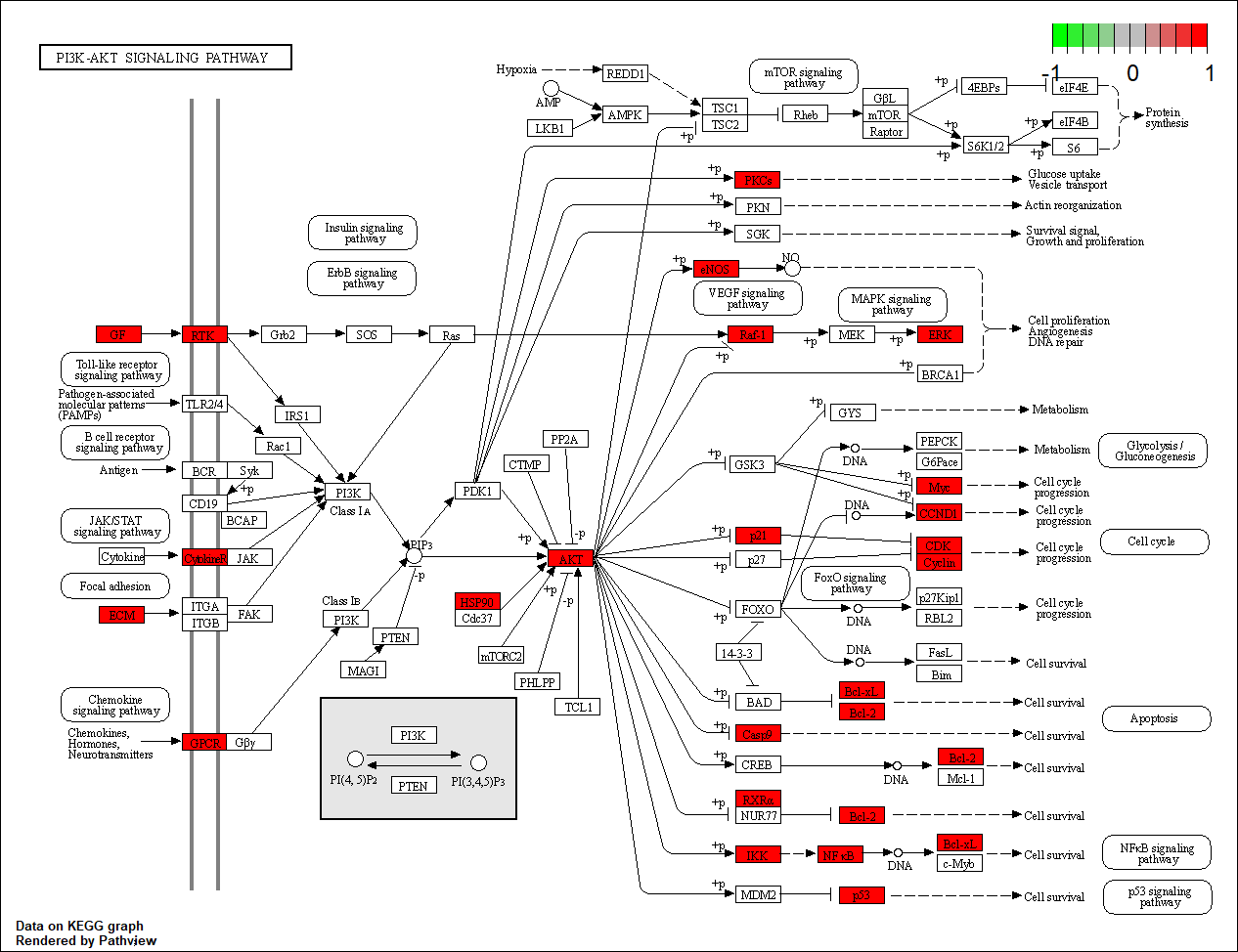

Supplement: Supplementary file 2 [file Image1.PNG]
